# Supplementary material for: Trends in reporting embolic and thrombotic events after COVID-19 vaccination: A retrospective, pharmacovigilance study
Source: PLoS One. 2022 Aug 1;17(8):e0269268. doi: 10.1371/journal.pone.0269268 (PMC9342794; doi:10.1371/journal.pone.0269268)
Supplement: Attachment — Submitted filename: Responses.docx [file pone.0269268.s006.docx]

**Response to Reviewer #1**

**Comment 1**

As the results of the study were based on ROR and data were self reported this should be obviously indicated in the title to Avoid misleading so I strongly suggest changing it to start with "Trends in reporting ETE after Covid 19 vaccination ...." instead of the term relationship.

**[Response]**

Thank you for the suggestion.

The title has been revised as follows to avoid misleading the reader. (P1, L1)

“Trends in reporting embolic and thrombotic events after COVID-19 vaccination: a retrospective, pharmacovigilance study”

**Response to Reviewer #2**

**Comment 1**

The abstract must be revised again to include the main performance evaluation results regarding the previous works.

**[Response]**

This manuscript addresses the fact that previous studies have reported only a small number of cases of ETEs. Therefore, we have revised the beginning of the Abstract as follows: (P1, L12)

“With the progression of global vaccination against coronavirus disease 2019 (COVID-19), embolic and thrombotic events (ETEs) following COVID-19 vaccination have been reported. To date, most reports on the type of COVID-19 vaccine and ETE have been based on clinical trials, and other reports include a small numbers of cases. Further, the relationship between the type of COVID-19 vaccine and ETE has not been clarified.”

**Comment 2**

Please make sure that all keywords have been used in the abstract and the title.

**[Response]**

We considered the following keywords in this manuscript: COVID-19 vaccine, Vaccine Adverse Event Reporting System (VAERS), embolic and thrombotic events (ETEs), and reported odds ratio (ROR).

We have added "reported odds ratio (ROR)" to the abstract and confirm that the other keywords are used in the abstract or title. (P1, L18)

“In this retrospective, pharmacovigilance study, we analyzed the Vaccine Adverse Event Reporting System (VAERS) reports from January 1, 2020 to June 18, 2021, and performed signal detection and time-to-onset analysis of adverse events by calculating the reported odds ratio (ROR) to understand ETE trends after COVID-19 vaccination based on the vaccine type.”

**Comment 3**

Figures still need to be amended, where the resolution is so bad which makes it difficult to read.

**[Response]**

Thank you for the suggestion.

We have revised Figure 3 to make it easier to read. All figures have been revised to the appropriate size and resolution using PACE (https://pacev2.apexcovantage.com/).

**Comment 4**

The conclusions section still need to revised to address the main contributions of the study to academics and practices, and recommendations of future works.

**[Response]**

We have reexamined the Conclusion section and revised it.

The following was added as a contribution to academics. (P20, L330)

“This study's methodology may complement a small number of post-vaccine adverse event reports, such as those from clinical trials, in post-vaccine adverse events that have a significant impact on patient quality of life.”

The following was added as a contribution to practice. (P20, L335)

“In addition, as it was suggested that the onset time of ETEs differed depending on the vaccine type, the vaccine recipients should be carefully monitored not only immediately after vaccination but also for a certain period thereafter.”

We have made the following revisions as recommendations for future research. (P20, L337)

“Further investigation, including the evaluation of ETEs stratified by age and sex, will provide more details on ETEs associated with COVID-19 vaccination. It is hoped that this will aid in taking precautions against ETEs associated with COVID-19 vaccination.”

**Comment 5**

A thorough proofreading is still required (best by a native English speaker).

**[Response]**

Thank you for your suggestion.

We have performed the native check again in accordance with your suggestion.
